# Sweet’s Syndrome Following Therapy with Hydroxychloroquine in a Patient Affected with Elderly-Onset Primary Sjogren’s Syndrome

**DOI:** 10.3390/medicines6040111

**Published:** 2019-11-15

**Authors:** Ciro Manzo, Nazareno Pollio, Maria Natale

**Affiliations:** 1 Rheumatology Outpatient Clinic, Poliambulatorio “Mariano Lauro”, Sant’Agnello—Distretto Sanitario 59 (Penisola Sorrentina), Azienda Sanitaria Locale Napoli 3 sud, Sant’Agnello, 80065 Naples, Italy; 2 Anatomy and Pathological Histology Department, University “Luigi Vanvitelli”, 80135 Naples, Italy; naznazxx@gmail.com; 3 Internal Medicine Department, Azienda Sanitaria Locale Napoli 3 sud, Gragnano, 80054 Naples, Italy; maria.natale.int@alice.it

**Keywords:** Sweet’s syndrome, neutrophilic dermatoses, hydroxychloroquine, primary Sjogren’s syndrome, adverse drug reaction, drug-induced syndrome, elderly patients

## Abstract

Sweet’s syndrome is an uncommon skin disease characterized by painful polymorphic lesions associated with fever and neutrophilia. When biopsied, these lesions reveal a diffuse infiltrate of mature neutrophils in the papillary dermis. Several drugs can induce Sweet’s syndrome (so-called drug-induced Sweet’s syndrome (DISS)) but reports of DISS associated with hydroxychloroquine (HCQ) are exceptionally limited. A 72-year-old Caucasian female patient with elderly-onset primary Sjogren’s syndrome (EOpSS) but low disease activity presented with an abrupt onset of painful nodular and papular erythematous skin lesions after two weeks of therapy with HCQ 400 mg. A histological examination revealed a diffuse infiltrate of mature neutrophils in the papillary dermis, without vasculitis. After therapy with 25 mg/day prednisone and HCQ withdrawal, the cutaneous manifestations disappeared. When prednisone was permanently discontinued, the primary Sjogren’s syndrome (pSS) manifestations worsened and therapy with HCQ 200 mg was reintroduced. In a few days, the same skin lesions reappeared. Withdrawal of HCQ and a new cycle of prednisone resulted in their permanent disappearance. We reported a case of DISS following therapy with HCQ in a female patient affected by EOpSS. According to a literature review, this is the first report of this association.

## 1. Introduction

Sweet’s syndrome (also known as “acute febrile neutrophilic dermatosis”) is a skin disease characterized by painful polymorphic lesions (for example, papules, nodules, or plaques), associated with fever and neutrophilia. When biopsied, these lesions reveal an infiltrate of mature neutrophils located in the upper or papillary dermis. The absence of vasculitis is mandatory for diagnosis.

Sweet’s syndrome belongs to the group of neutrophilic dermatoses (NDs), a heterogeneous group of dermatoses (such as pyoderma gangrenosum, hidroadenitis suppurativa, erythema elevatum diutinum, subcorneal pustular dermatosis), in which histological examination shows intense infiltrate of mature neutrophils with no evidence of infection. At present, NDs are considered to be polygenic auto-inflammatory diseases, characterized by mutations in so-called “inflammasomes”, which induce excessive inflammatory cytokine production (mostly IL-1) by innate immune cells in response to danger signals. Fever and extra cutaneous manifestations can be present in all NDs. Leukocytoclastic vasculitis can be present in some NDs, but not in Sweet’s syndrome. 

A whole series of extra-cutaneous manifestations has been described in patients with Sweet’s syndrome, but in the majority of cases it is very difficult to determine whether they are just a coincidence, or if they are strictly linked to skin manifestations [1,2]. 

Since its first description in 1964 by Robert Douglas Sweet [3], several hundred cases have been published [4,5]. As of today, epidemiologic information, including incidence, is unknown. There are three different classifications, namely, classical, malignancy-associated, and drug-induced (so-called drug-induced Sweet’s syndrome (DISS)). In particular, several drugs are associated with Sweet’s syndrome, with anticancer agents and granulocyte-colony stimulating factor (GCSF) on the front line [1,6,7,8]. So far, exceptionally limited instances of Sweet’s syndrome following therapy with hydroxychloroquine (HCQ) have been reported [9].

Sjogren’s syndrome (SS) is an autoimmune disease characterized by chronic lymphocytic infiltration of exocrine glands, leading to sicca syndrome. Several different internal organs may also be affected, causing systemic impairment. SS may occur alone as primary SS (pSS) or in association with other autoimmune disorders as secondary SS (sSS). pSS is considered one of the most common autoimmune rheumatic diseases and is more frequent in women (female/male ratio of 9:1) [10]. Factors that trigger the pathophysiological events in pSS include genetic predisposition (for genes encoding human leucocyte antigen (HLA)-B8, HLA-Dw3, HLA-DR3, and HLA-DRw52, infections (especially Epstein–Barr virus), environmental factors such as ultraviolet radiation, and hormonal disorders. Epithelial damage is the first step in the pathogenetic sequence with the subsequent release of autoantigens, the activation of innate and acquired immunity, and the production of autoantibodies. Among these, antibodies against small ribonucleoproteins SS-A/Ro and SS-B/La are particularly important [11]. 

At the end of 2016, the American College of Rheumatology (ACR) and the European League Against Rheumatism (EULAR) jointly presented new pSS criteria. These criteria apply to patients who have at least one symptom of ocular or oral dryness. Of all the laboratory tests, diagnostic importance was attributed only to SSA/Ro autoantibodies (3 points). The ocular staining score and Schirmer’s test were retained as part of the assessment of lachrymal gland function (1 point), while the measurement of unstimulated salivary flow was proposed for the assessment of salivary gland function (1 point). Histopathological examination of minor salivary gland biopsies with the assessment of the so-called “focus score” (FS) of infiltrate cells remained an important element of diagnosis (FS > 1 gives 3 points). According to ACR/EULAR criteria, a diagnosis is made if a patient has a sum of ≥4 points, but it must be noted that a cut-off of 5 points (instead of 4) raises the specificity of these criteria from 89% to 98% [12,13].

The EULAR Sjögren’s Syndrome Disease Activity Index (ESSDAI) is a clinical index that measures disease activity in pSS by evaluating 12 different domains, with each domain divided into three or four levels of activity and scores ranging from 0 to 123. A score of 5 indicates moderate disease activity, whereas a score of >13 indicates high activity [14].

According to the definition, elderly-onset pSS (EOpSS) affects people older than 60 [15]. EOpSS has an estimated overall prevalence of approximately 3%, but epidemiological data are very heterogeneous, depending on variables such as geographic area and diagnostic criteria. As an example, in an Italian cohort, 6% of patients had EOpSS [16], while in a Tunisian cohort this value was 30% [17]. 

## 2. Case Report

**Figure 1:**
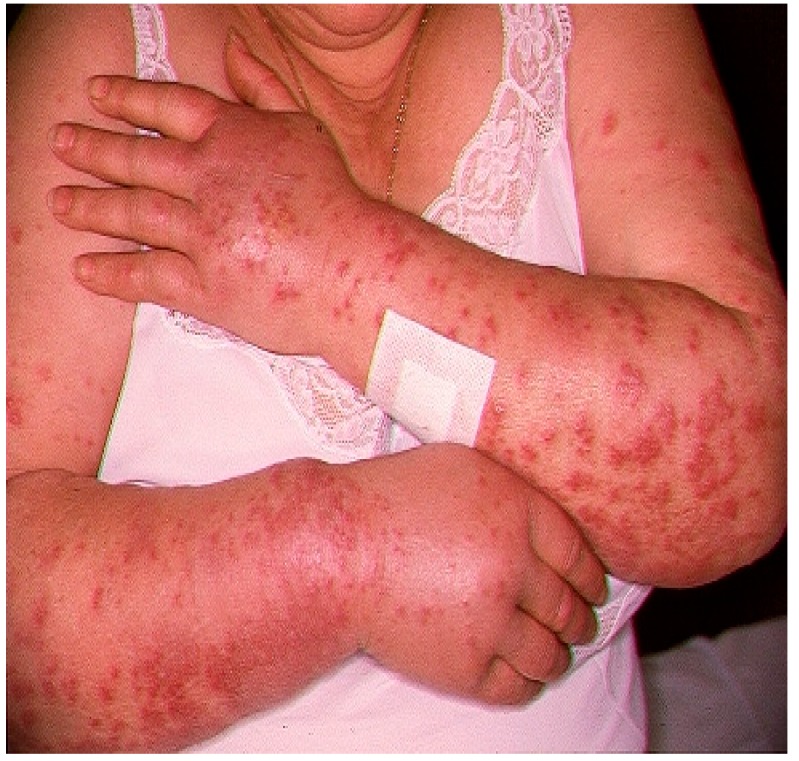
Papulonodular manifestations in our patient, following therapy with hydroxychloroquine (HCQ) (400 mg/day).

RA was a 72-year-old Caucasian woman affected by pSS (score = 7 according to the ACR/EULAR criteria) with low disease activity (ESSDAI = 4). Her weight was 76 kilograms with a body mass index equal to 26.93 kg/mg^2^. Her comorbidities included arterial hypertension, for which she took amlodipine 5 mg daily, and non-hemodynamically significant carotid atheromasia, for which she took acetylsalicylic acid 100 mg at lunchtime. Two weeks after the introduction of HCQ 400 mg in two daily doses, an abrupt onset of painful papulonodular skin lesions occurred on her upper limbs (Figure 1), which was associated with general malaise and fever (T = 38.5 °C).

**Figure 2:**
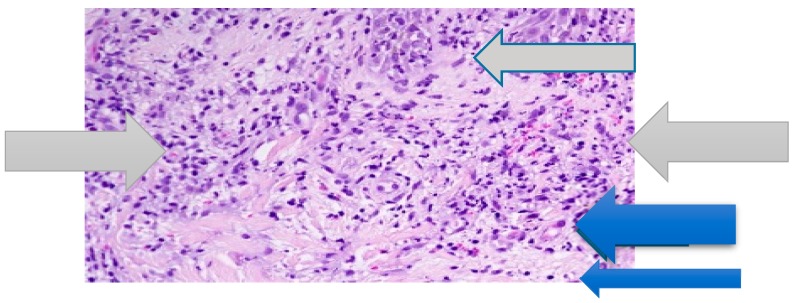
Papillary dermal edema, swollen endothelial cells (blue arrow), and diffuse infiltrate of neutrophils (grey arrow). No evidence of vasculitis (hematoxylin and eosin stain, ×100 magnification).

The patient consulted a dermatologist who proposed therapy with an anti-histamine, with no improvement. The laboratory tests showed the following: erythrocyte sedimentation rate (ESR) = 38 mm/h; C-reactive protein (CRP) concentration = 12 mg/dL (normal value <6); leukocytes = 12.000/mmc (normal value <8000/mmc) with segmented neutrophils = 8500/mmc (>70% of total). The other laboratory data were in the normal ranges. In particular, hemoglobin was equal to 13.1 g/dL (normal value >12.0 g/dL); transaminases, creatine phosphokinase (CPK), protein electrophoresis, rheumatoid factor (RF), antinuclear cytoplasmic antibodies (ANCA), and total serum immunoglobulin E (IgE) levels were in their normal ranges. Urine cultures and urine blood tests were negative and fecal calprotectin dosage was in the normal range. A histological examination revealed diffuse infiltrate of mature neutrophils in the papillary dermis with vasculitis absent (Figure 2). 

According to the criteria proposed by von der Driesch in 1994 [18], classical Sweet’s syndrome was diagnosed. According to these criteria, the presence of both major criteria and two of the four minor criteria is required in order to diagnose this syndrome (Table 1). 

Therapy with prednisone (25 mg/day) was proposed. Cutaneous manifestations improved, but as soon as the dosage of prednisone was reduced, they quickly reappeared. Since HCQ was recently used by the patient, the dermatologist hypothesized that HCQ may be involved and advised the patient to withdraw it. The cutaneous manifestations disappeared completely, and the patient stopped the prednisone. However, when the prednisone was permanently discontinued, the pSS manifestations worsened and the rheumatologist reintroduced therapy with HCQ 200 mg. In a few days, the same skin lesions reappeared. Withdrawal of HCQ and a new cycle of prednisone allowed for the permanent disappearance of the skin lesions. 

## 3. Literature Review

A comprehensive literature search was carried out using the MEDLINE and EMBASE bibliographic databases. The following main search terms were used: Sweet’s syndrome, primary Sjogren’s syndrome, Sjogren’s syndrome, secondary Sjogren’s syndrome, drug-induced Sweet’s syndrome, hydroxychloroquine, Sweet’s syndrome AND Sjogren’s syndrome. The search was made on 11 October 2019. References from all of the selected studies were also examined.

The search terms returned nine articles which are listed in Table 2. 

Sweet’s syndrome was associated with pSS in only one case [22], while in all the other cases its association was with sSS (in particular, SLE, rheumatoid arthritis, Crohn’s disease, and myelodysplastic syndrome were associated diseases). The two cases reported by Bodard et al. were the only ones where Sweet’s syndrome followed therapy with HCG in two young women, one who was affected by SLE and the second who was affected by SS associated with SLE [9]. 

## 4. Discussion

Sweet’s syndrome is an uncommon skin disease with a challenging diagnosis. As already highlighted, Sweet’s syndrome may be drug-induced. The most commonly reported drugs are GCSF and anticancer agents, such as hypomethylating agents, imatinib mesylate, and lenalidomide. The association with other drugs (e.g., diuretics, non-steroidal anti-inflammatory agents, antibiotics) is anecdotal. 

In 1996, Walker and Cohen proposed five diagnostic criteria for DISS (Table 3) [27]. All five criteria must be present for diagnosis. As of today, these proposed criteria are still considered valid. 

HCQ is a synthetic antimalarial drug derived from 4-aminoquinoline and has been widely used for several decades for the treatment of several inflammatory and autoimmune rheumatic diseases. In pSS, some clinical trials highlighted its utility in patients with low disease activity (ESSDAI score of <5), showing improvement in fatigue, joint pain, and some laboratory findings (such as hypergammaglobulinemia, ESR, and RF levels) [28,29]. 

A dosage between 3 and 6 mg/bodyweight/day is considered therapeutic, but in obese individuals, the dosage must be assessed considering the patient’s ideal bodyweight. Usually, an initial attack dose of 400 mg/day (divided in two doses) is proposed, followed by a maintenance dose of 200 mg (even for life). Due to the high affinity of HCQ for melanin, its highest concentration is in the skin (epidermis values are much higher than in the dermis) and in the eye [30]. 

There are numerous side effects during HCQ therapy, some of which are favored according to predisposing or precipitant factors. Among these, cytochrome P450 3A4 inhibitors (such as indinavir, nelfinavir, ritonavir, clarithromycin, erythromycin, fluconazole, itraconazole, ketoconazole, amiodarone, cimetidine, diltiazem, and verapamil) may increase the half-life of HCQ, favoring its toxicity [31]. 

Xerosis and hyperpigmentation in the skin and mucous membranes are the most common skin side effects, while urticarial lesions, maculopapular rashes, and erythroderma are very uncommon [32]. As already highlighted, one case of DISS related to HCQ was recently reported in a young female patient affected by Sjogren’s syndrome associated with SLE [9]. 

When we applied the Naranjo scale, our patient had a score of 10 (see Table 4). The Naranjo scale, also called the “Naranjo Adverse Drug Reaction Probability Scale”, was developed in 1981 by Naranjo and colleagues to standardize causality assessments. Its score results in a significant increase in inter- and intra-rater agreement compared with global introspection alone. The sum of the scores from ten questions ranges from −4 to +13 and is interpreted to reflect the strength of the probability that a drug has caused an adverse drug reaction (ADR) rather than the complication being a manifestation of the disease. A score of greater than nine is empirically defined as “definitely” having caused the ADR; a score of five to eight indicates the drug “probably” caused the ADR; a score of one to four indicates the drug “possibly” caused the ADR; and a score of less than one indicated a “doubtful” association with the drug [33].

As the literature review highlighted, Sweet’s syndrome rarely occurs in sSS patients, and its association with pSS is exceptionally limited. As of today, the reasons why DISS was caused after therapy with HCQ in our patient are only speculative. Some investigators hypothesized that an inciting stimulus, such as an antigen in an individual with a genetic predisposition, likely creates a pro-inflammatory state [4]. This general pathogenic model may also apply to HCQ-induced Sweet’s syndrome. HCQ tropism for neutrophils may be an adjunctive favoring factor [30,32]. Our patient took no cytochrome P450 3A4 inhibitors. Finally, variability in the extent of absorption may lead to differences in steady-state HCQ concentrations among patients, potentially contributing to variabilities in responses and side effects [34]. The lack of some data (e.g., detection of HCQ levels in the blood and in the papillary dermis or genetic evaluations) could be a limitation of our case report, but the exceptional nature of the association we reported must be highlighted. 

## 5. Conclusions

Sweet’s syndrome is an uncommon skin disease, and its association with pSS and HCQ has been very rarely reported. 

We reported a case of DISS following therapy with HCQ in an elderly woman affected by pSS. According to the literature review, this case is the first such report of this association. 

## Figures and Tables

**Table 1:** Classical Sweet’s syndrome: Proposed diagnostic criteria.

**Major Criteria**
1. Abrupt onset of tender or painful erythematous plaques or nodules.
2. Neutrophilic infiltration in the dermis without leukocytoclastic vasculitis.
**Minor Criteria**
1. Preceded by a nonspecific respiratory or gastrointestinal tract infection or vaccination or associated with: (a) inflammatory diseases such as chronic autoimmune disorders; (b) infections; (c) pregnancy; (d) hemoproliferative diseases or solid malignant tumors.
2. General malaise and fever (>38 °C).
3. Laboratory values during onset: Raised erythrocyte sedimentation rate (ESR) value of >20 mm; C-reactive protein positive; >70% segmented neutrophils in peripheral blood smear; leukocytosis value of >8000/mmc (3 of 4 of these values are necessary).
4. Fast response to treatment with systemic corticosteroids or potassium iodide.

**Table 2:** Sweet’s syndrome, Sjogren’s syndrome, and hydroxychloroquine: Case reports in the indexed literature.

First Author	Year of Publication	pSS	HCG	sSS	Sweet’s Syndrome?
Levenstein MM [19]	1991	NO	NO	YES	Probably no. Annular erythema.
Bianconcini G [20]	1991	NO	NO	YES	Unclear data
Osawa H [21]	1997	NO	NO	YES	YES
Vatan R [22]	1997	YES	NO	NO	YES
Harada Y [23]	2001	NO	NO	YES	YES
Foster EN [24]	2005	NO	NO	YES	YES
Souissi A [25]	2007	NO	NO	YES	YES
Mrabet D [26]	2011	NO	NO	YES	YES
Bodard Q [6]	2019	NO	YES	YES	YES

pSS—primary Sjogren’s syndrome; HCG—hydroxychloroquine; sSS—secondary Sjogren’s syndrome.

**Table 3:** Diagnostic criteria for drug-induced Sweet’s syndrome (DISS) proposed by Walker and Cohen.

1. Acute onset of painful erythematous skin disease;
2. Dermal neutrophilic infiltrate without evidence of vasculitis on histopathological examination;
3. Fever (temperature >38 °C);
4. Temporal relationship between drug and clinical manifestations or temporal-related recurrence after drug challenge;
5. Temporal-related resolution of skin lesions after drug withdrawal or treatment with systemic corticosteroids.

**Table 4:** The Naranjo scale questions and weighted scores in our patient.

	Yes	No	Do Not Know	Score
1. Are there previous *conclusive* reports on this reaction?	+1	0	0	+1
2. Did the adverse event appear after the suspected drug was administered?	+2	−1	0	+2
3. Did the adverse reaction improve when the drug was discontinued or a *specific* antagonist was administered?	+1	0	0	+1
4. Did the adverse reaction reappear when the drug was readministered?	+2	−1	0	2
5. Are there alternative causes (other than the drug) that could on their own have caused the reaction?	−1	+2	0	+2
6. Did the reaction reappear when a placebo was given?	−1	+1	0	0
7. Was the drug detected in the blood (or other fluids) in concentrations known to be toxic?	+1	0	0	0
8. Was the reaction more severe when the dose was increased, or less severe when the dose was decreased?	+1	0	0	1
9. Did the patient have a similar reaction to the same or similar drugs in *any* previous exposure?	+1	0	0	0
10. Was the adverse event confirmed by any objective evidence?	+1	0	0	1
	**Total**	**Score**		**10**

## References

[1] Cohen P.R. (2007). Sweet’s syndrome—A comprehensive review of an acute febrile neutrophilic dermatosis. Orphanet J. Rare Dis..

[2] Marzano A.V., Ortega-Loayza A.G., Heath M., Morse D., Genovese G., Cugno M. (2019). Mechanisms of Inflammation in Neutrophil-Mediated Skin Diseases. Front. Immunol..

[3] Sweet R.D. (1964). An acute febrile neutrophilic dermatosis. Br. J. Dermatol..

[4] Heath M.S., Ortega-Loayza O. (2019). Insights into the pathogenesis of Sweet’s syndrome. Front. Immunol..

[5] Lopes Cacola R., Soares M., Cardoso C., Furtado A. (2016). Seet’s syndrome complicating ulcerative cfolitis: A rare association. BMJ Case Rep..

[6] Lund J.J., Stratman E.J., Jose D., Xia L., Wilson D., Moizuddin M. (2010). Drug-induced bullous Seet syndrome with multiple autoimmune features. Autoimmune Dis..

[7] Arun Kumar A.U., Elsayed M.E., Alghali A., Ali A.A., Mohamed H., Hussein W., Hackett C., Leonard N., Stack A.G. (2018). Sweet syndrome: A rare feature of ANCA-associated vasculitis or unusual consequence of azathioprine-induced treatment. Allergy Asthma Clin. Immunol..

[8] Kasirye Y., Danhof R.S., Epperla N.E., Garcia-Montilla R.J. (2011). Sweet’s syndrome: One disease, multiple faces. Clin. Med. Res..

[9] Bodard Q., Carre D., Chenal P., Zarnitsky C., Midhat M., Litrowski N. (2019). Drug-induced related to hydroxychloroquine: About 2 cases. Rev. Med. Interne.

[10] Chen X., Wu H., Wei W. (2018). Advances in the diagnosis and treatment of Sjogren’s syndrome. Clin. Rheumatol..

[11] Maślińska M., Przygodzka M., Kwiatkowska B., Sikorska-Siudek K. (2015). Sjögren’s syndrome: Still not fully understood disease. Rheumatol. Int..

[12] Shiboski C.H., Shiboski S.C., Seror R., Criswell L.A., Labetoulle M., Lietman T.M., Rasmussen A., Scofield H., Vitali C., Bowman S.J. (2017). 2016 American College of Rheumatology/European League Against Rheumatism classification criteria for primary Sjögren’s syndrome: A consensus and data-driven methodology involving three international patient cohorts. Ann. Rheum. Dis..

[13] Franceschini F., Cavazzana I., Andreoli L., Tincani A. (2017). The 2016 classification criteria for primary Sjogren’s syndrome: what’s new?. BMC Med..

[14] Seror R., Bowman S.J., Brito-Zeron P., Theander E., Bootsma H., Tzioufas A., Gottenberg J.-E., Ramos-Casals M., Dorner T., Ravaud P. (2015). EULAR Sjögren’s syndrome disease activity index (ESSDAI): A user guide. RMD Open.

[15] Manzo C., Maslinska M. (2018). Primary Sjögren’s syndrome in the elderly: Does age of onset make a difference?. EMJ Rheumatol..

[16] Botsios C., Furlan A., Ostuni P., Sfriso P., Andretta M., Ometto F., Raffeiner B., Todesco S., Punzi L. (2011). Elderly onset of primary Sjögren’s syndrome: Clinical manifestations, serological features and oral/ocular diagnostic tests. Comparison with adult and young onset of the disease in a cohort of 336 Italian patients. Jt. Bone Spine.

[17] Chebbi W., Ben Salem W., Klii R., Kessomtini W., Jerbi S., Sfar M.H. (2015). Primitive Sjögren syndrome in the elderly: Clinical and immunological characteristics. Pan Afr. Med. J..

[18] Von den Driesch P. (1994). Sweet’s syndrome (acute febrile neutrophilic dermatosis). J. Am. Acad. Dermatol..

[19] Levenstein M.M., Fisher B.K., Fisher L.L., Pruzanski W. (1991). Simultaneous occurrence of subacute cutaneous lupus erythematosus and Sweet syndrome. A marker of Sjögren syndrome?. Int. J. Dermatol..

[20] Bianconcini G., Mazzali F., Candini R., Dallasta A., Gobbi F. (1991). Sweet’s syndrome (acute febrile neutrophilic dermatosis) associated with Sjögren’s syndrome. A clinical case. Minerva Med..

[21] Osawa H., Yamabe H., Seino S., Fukushi K., Miyata M., Inuma H., Kaizuka M., Tamura N., Tsunoda S., Baba Y. (1997). A case of Sjögren’s syndrome associated with Sweet’s syndrome. Clin. Rheumatol..

[22] Vatan R., Sire S., Constans J., Ragnaud J.M. (1997). Association of primary Gougerot-Sjögren syndrome and Sweet syndrome. Apropos of a case. Rev. Med. Interne.

[23] Harada Y., Egi Y., Honda Y., Shirota T., Hayashi T. (2001). Multiple myeloma with Sweet disease developing from monoclonal gammopathy of undetermined significance and Sjögren syndrome. Rinsho Ketsueki.

[24] Foster E.N., Nguyen K.K., Sheikh R.A., Prindiville T.P. (2005). Crohn’s disease associated with Sweet’s syndrome and Sjögren’s syndrome treated with infliximab. Clin. Dev. Immunol..

[25] Souissi A., Benmously R., Fenniche S., Zarrouk M., Marrek H., Debbiche A., Ayed M.B., Mokhtar I. (2007). Sweet’s syndrome: A propos of 8 cases. Tunis. Med..

[26] Mrabet D., Saadi F., Zaraa I., Chelly I., Sahli H., Osmane A.-B., Meddeb N., Sellami S. (2011). Sweet’s syndrome in a patient with rheumatoid arthritis, Sjögren’s syndrome and lymph node tuberculosis. BMJ Case Rep..

[27] Walker D.C., Cohen P.R. (1996). Trimethoprim-sulfamethoxazole-associated acute febrile neutrophilic dermatosis: Case report and review of drug-induced Sweet’s syndrome. J. Am. Acad. Dermatol..

[28] Wang S.Q., Zhang L.W., Hua H. (2017). Is hydroxychloroquine effective in treating primary Sjogren’s syndrome: A systematic review and meta-analysis. BMC Musculoskelet. Disord..

[29] Ktulze A.A., Hene R.J., Kallenberg C.G., van Bijsterveld O.P., van der Heide A., Kater L., Bijlsma J.W. (1993). Hydroxychloroquine treatment for primary Sjogren’s syndrome: A two year double blind crossover trial. Ann. Rheum. Dis..

[30] Haladyi E., Sikora M., Felis-Giemza A., Oleslnka M. (2018). Antimalarials. Are they effective and safe in rheumatic diseases?. Reumatologia.

[31] Manzo C., Gareri P., Castagna A. (2017). Psychomotor agitation following treatment with hydroxychloroquine. Drug Saf. Case Rep..

[32] Skare T., Ferrari Ribeiro C., Souza F.H.M., Haendchen L., Jordao J.M. (2010). Antimalarials cutaneous side effects:a study in 209 users. Cutan. Ocul. Toxicol..

[33] Naranjo C.A., Busto U., Sellers E.M., Sandor P., Ruiz I., Robert E.A., Janecek E., Domecq C., Greenblatt D.J. (1981). A method for estimating the probability of adverse drug reaction. Clin. Pharmacol. Ther..

[34] Tett S.E., Cutler D.J., Day R.O., Brown K.F. (1989). Bioavailability of hydroxychloroquine tablets in healthy volunteers. Br. J. Clin. Pharmacol..

